# Effects of probiotics on serum levels of Th1/Th2 cytokine and clinical outcomes in severe traumatic brain-injured patients: a prospective randomized pilot study

**DOI:** 10.1186/cc10579

**Published:** 2011-12-02

**Authors:** Min Tan, Jing-Ci Zhu, Jiang Du, Li-Mei Zhang, Hua-Hua Yin

**Affiliations:** 1 Department of Nursing, Affiliated Hospital of North Sichuan Medical College, 63 Wenhua Road, Nanchong 637000, Sichuan, China; 2 School of Nursing, Third Military Medical University, 30 Gaotanyan Street, Chongqing 400038, China; 3 Department of General Surgery, Affiliated Hospital of North Sichuan Medical College, 63 Wenhua Road, Nanchong 637000, Sichuan, China

## Abstract

**Introduction:**

Traumatic brain injury (TBI) is associated with a profound immunological dysfunction manifested by a severe shift from T-helper type 1 (Th1) to T-helper type 2 (Th2) response. This predisposes patients to infections, sepsis, and adverse outcomes. Probiotic bacteria have been shown to balance the Th1/Th2 cytokines in allergic murine models and patients. For the present study, we hypothesized that the enteral administration of probiotics would adjust the Th1/Th2 imbalance and improve clinical outcomes in TBI patients.

**Methods:**

We designed a prospective, randomized, single-blind study. Patients with severe TBI and Glasgow Coma Scale scores between 5 and 8 were included, resulting in 26 patients in the control group and 26 patients in the probiotic group. All patients received enteral nutrition via a nasogastric tube within 24 to 48 hours following admission. In addition, the probiotic group received 10^9 ^bacteria of viable probiotics per day for 21 days. The associated serum levels of Th1/Th2 cytokines, Acute Physiology and Chronic Health Evaluation (APACHE) II and Sequential Organ Failure Assessment (SOFA) scores, nosocomial infections, length of ICU stay, and 28-day mortality rate were studied.

**Results:**

The patients responded to viable probiotics, and showed a significantly higher increase in serum IL-12p70 and IFNγ levels while also experiencing a dramatic decrease in IL-4 and IL-10 concentrations. APACHE II and SOFA scores were not significantly affected by probiotic treatment. Patients in the probiotic group experienced a decreased incidence of nosocomial infections towards the end of the study. Shorter ICU stays were also observed among patients treated with probiotic therapy. However, the 28-day mortality rate was unaffected.

**Conclusions:**

The present study showed that daily prophylactic administration of probiotics could attenuate the deviated Th1/Th2 response induced by severe TBI, and could result in a decreased nosocomial infection rate, especially in the late period.

**Trial registration:**

ChiCTR-TRC-10000835.

## Introduction

Traumatic brain injury (TBI) is associated with profound immunological dysfunction. Severe immunodepression involving the cell-mediated immune response may occur early and may even last for several weeks [1]. In addition, hyperinflammation may also occur as a result of injury. This can be attributed to the production of glucocorticoids after brain injury, which inhibits the production of IL-12, a cytokine secreted by monocytes/macrophages and dendritic cells (DCs). IL-12 plays a critical role in driving naive CD4^+ ^T-helper cells (Th0) to T-helper type 1 (Th1) subtypes, directly contributing to impaired cellular immunity [2]. IL-4 and IL-10, two anti-inflammatory T-helper type 2 (Th2) cytokines known for their ability to suppress the production of IFNγ and IL-2 by Th1 cells [3], appear in high concentrations within hours following the brain injury [2,4,5]. An obvious shift from Th1 response to Th2 is presented, and renders patients more vulnerable to infections, sepsis, and adverse outcomes [4,5]. The incidence of ventilator-associated pneumonia (VAP) can reach 60% in this category of patients [2]. When we manage postinjury infectious complications with antibiotics, therefore, it is believed that some therapeutic strategies aimed at enhancing intrinsic immune functions are also needed. In particular, the rapid emergence of antibiotic-resistant pathogens as well as the lack of new antimicrobial agents needs to be taken into account in the near future [6,7].

Probiotics have increasingly been shown to manage various infectious diseases due to their ability to restore the nonpathogenic digestive flora that commonly disappear as a result of various diseases or following medical treatment with pharmaceuticals such as antibiotics [8]. Research indicates that probiotics reduce the growth of potentially pathogenic microorganisms, improve gut mucosal barrier function, and modulate local and systemic immune functions [9]. Data support the use of probiotics as a treatment for infectious diarrhea, antibiotic-associated diarrhea, and necrotizing enterocolitis [9]. However, the results of limited studies that focused on critically ill patients remain controversial.

Two recent meta-analysis of probiotics in critically ill patients achieved different conclusions: one demonstrated the efficacy of probiotics on the incidence of VAP [10], while the other concluded that probiotics had no effects on these patients [11]. Schultz and Haas explained that the two following reasons might contribute to the difference between the two meta-analysis [12]: first, the meta-analysis that achieved negative results included trials of postoperative patients who are often admitted to the ICU for too short a stay to develop VAP [11]; and second, this meta-analysis did not include one important trial that showed a reduced rate of VAP with probiotics [13]. Morrow and Kollef concluded that much of the current confusion surrounding the efficacy of probiotics stemmed from the heterogeneity of study populations, differences in study designs, inconsistencies in the strains used, and the limited understanding of probiotics' mechanisms of action [14]. Although the immunomodulatory effects of probiotics have been studied widely *in vitro *and in animal models [15], it is difficult to extrapolate the results of animal studies to human populations. More well-designed studies are therefore recommended that incorporate both clinical outcomes and measurement of biomarkers putatively related to clinical effects such as immune markers, changes in the microbiota, and gut barrier function, in order to generate data that achieve more consistent evidence [9].

To date, no study has focused on the effects of probiotics in severe traumatic brain-injured patients who are at extremely high risk of VAP. We therefore conducted a prospective randomized trial to investigate the influence of probiotics on this specific category of patients. The primary endpoint was to determine whether and to what extent probiotics affect the Th1/Th2 cytokines. Our secondary endpoints were the infection rate, use of antibiotics, ICU length of stay, and 28-day mortality rate.

## Materials and methods

The present study was conducted between October 2009 and January 2011 at the Affiliated Hospital of North Sichuan Medical College (Nanchong, Sichuan, China), in a six-bed specialized ICU in the Department of Neurosurgery. The study protocol was approved by the Human Ethics Committee of the hospital (AHNSMC, 2009066) and was performed in compliance with the Helsinki Declaration. Due to the unconsciousness of patients, informed consent was obtained from their closest family members prior to their admittance into the study. Further consent was not obtained from patients after they recovered, as this was not required by the Human Ethics Committee.

The following inclusion criteria were applied to patients: closed head injury alone; admission within 24 hours after trauma; a Glasgow Coma Scale score between 5 and 8; aged 18 to 60 years old; and able to be fed via nasogastric tube within 48 hours after admission. Exclusion criteria were: previous significant digestive, hematological, and endocrine diseases; immunosuppression; presence of pneumonia or other infectious diseases upon admission; HIV-positive; other associated trauma such as extremity fractures and chest or abdominal trauma; cancer; pregnancy, or lactation; and obesity (body mass index > 30) or malnutrition (body mass index < 18.5).

After inclusion (which was done within 48 hours after admission), patients were randomized with a ratio of 1:1 into the probiotic group or the control group. Randomization was done with a computer-generated random number and sealed envelopes kept by a person not involved in the investigation. The enrolled patients, those who processed samples, and the bedside nurses in the research unit were blind to the study design. The investigators as well as the physicians in charge knew whether or not the patient had received probiotics.

All patients received enteral nutrition (EN) (3.8 g protein, 13.8 g carbohydrate, 3.4 g fat/100 ml, osmolarity 250 mOsm/l, no fibers; Ruisu, Huarui Pharmaceutical Co., Ltd, Beijing, China) within 48 hours following hospital admission by nasogastric tube, starting at 10 kcal/kg/day and gradually increasing to an energy target of 30 kcal/kg body weight/day and 0.2 gN/kg body weight/day on day 3. A rate of 25 ml/hour was initiated and then increasingly progressed by 25 ml/hour every 4 hours until a goal of 125 ml/hour was achieved. If the gastric residual volumes exceeded 150 ml after a second check using a 60-ml syringe, prokinetic agents were initiated and feeds were resumed and advanced until the goal rate was achieved. If the patient was unable to meet the energy goal after 5 days by enteral route alone, a supplementation of parenteral nutrition was initiated. If the patient transitioned to parenteral nutrition and could not resume enteral feeding for more than 2 days, the study was discontinued prematurely.

The probiotic group received seven sachets of viable probiotics three times a day, at 7:00, 15:00, and 23:00 hours, providing a total of 10^9 ^bacteria. Each sachet of probiotics (Golden Bifid, Shuangqi Pharmaceutical Co., Ltd, Inner Mongolia, China) contained 0.5 × 10^8 ^*Bifidobacterium longum*, 0.5 × 10^7 ^*Lactobacillus bulgaricus*, and 0.5 × 10^7 ^*Streptococcus thermophilus*. The probiotics were dissolved in 20 ml sterilized distilled water and administered through a nasogastric tube for 21 consecutive days. Pre and post administration of the drug, an additional 40 to 60 ml sterile water was given to flush the tube. When patients started oral intake and the nasogastric tube was removed, probiotics were ingested orally for the remaining days. Study participants were not allowed to use any other product containing probiotics during the trial. Another conventional therapy for these patients included operation when necessary, antibiotics, using mannitol to reduce intracranial pressure and oxygen absorption, and so forth. Tracheotomy was performed when patients presented complications related to dyspnea or hypoxemia, produced too much thick sputum to aspirate via nasal suctioning, or were expected to remain in a long period of coma. Intravenous pantoprazole (40 mg/day) was administered as a stress ulcer prophylaxis (generally for 3 to 4 days from admission) if deemed necessary by the attending physician.

Samples for complete blood count, blood gases, liver and renal function, and C-reactive protein (CRP) were collected in the morning on days 1 (before the initiation of EN), 4, 8, 15, and 21, and were analyzed at the hospital clinical chemistry laboratory. Combined with clinical data recorded by nurses and physicians in charge, the Acute Physiologic Chronic Health Evaluation (APACHE) II scores as well as the Sequential Organ Failure Assessment (SOFA) scores were evaluated [16,17]. The day when EN initiated was designated day 1. Serum samples separated from the whole blood by centrifugation were also collected at the same time points and were stored at -80°C until cytokine analysis was performed. Sera from 20 age-matched and sex-matched healthy volunteers were collected to serve as normal controls. Serum levels of IL-12p70, IL-4, IL-6, IL-10 and IFNγ were determined using the human ELISA kits of R&D Systems, Inc. (Minneapolis, MN, USA) according to instructions given by the manufacturer. All determinations were performed in duplicate and the mean of two observations was applied. The minimum detectable doses of IL-12p70, IL-4, IL-6, IL-10 and IFNγ with these assay kits were 15, 4, 1, 4 and 4 pg/ml, respectively.

Nosocomial infections were prospectively recorded by the physicians in charge of the patient according to the Centers for Disease Control and Prevention criteria [18]. Specifically, VAP was defined as pneumonia occurring more than 48 hours after endotracheal intubation, and was diagnosed by the presence of both a new or progressive radiographic infiltrate plus at least two clinical features - fever > 38.0°C, leucocytosis (white blood cells count > 12 × 10^9^/l), leucopenia (white blood cells count < 4 × 10^9^/l), or purulent tracheobronchial secretions - and positive semiquantitative cultures of tracheobronchial secretions [19]. Surveillance cultures of tracheobronchial secretions were taken once or twice weekly depending on patients' clinical symptoms. Multiple infections in the same patient were deemed to be one endpoint. The use of antibiotics, the ICU length of stay, as well as the 28-day mortality rate were also recorded. The ICU length of stay was defined as the number of days from the day that EN started to the discharge of the patient from the ICU.

### Statistical analysis

Because no previous study had examined the effect of probiotics in this context, we estimated that a sample size of 40 patients would be sufficient for this study. Based on previous experiences and studies, it was estimated that there would be a 30% dropout after randomization. We aimed to recruit 52 patients in total and analyze data per protocol as well as by intention to treat. Per-protocol analysis was interpreted to include only patients who participated in the trial for 21 days.

The data were analyzed using the statistical software program SPSS 11.5 (SPSS Inc., Chicago, IL, USA). The Shapiro-Wilk test was used to assess whether continuous data were normally distributed. For continuous variables, differences between groups were tested with Student's *t *test or analysis of variance for normally distributed data or the Mann-Whitney *U *test for non-normally distributed data. Comparisons of categorical data were done by chi-squared test or Fisher's exact test. Two-sided *P *< 0.05 was considered statistically significant.

## Results

**Figure 1:**
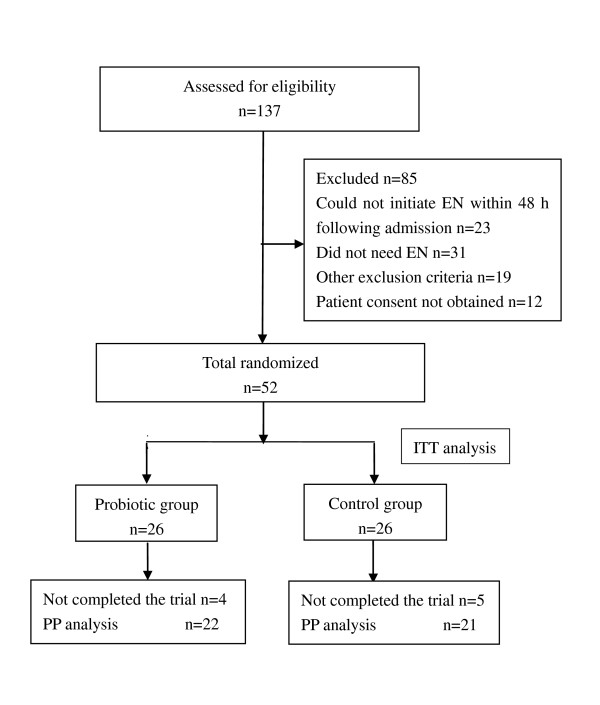
**CONSORT flow diagram of the trial**. EN, enteral nutrition; ITT, intention to treat; PP, per protocol.

During the 15-month study period, 137 patients were assessed for eligibility and 52 were enrolled. After randomization, three patients in the probiotic group died on days 4, 3, and 16, respectively; five of the controls died on days 24, 10, 10, 10, and 4, respectively; and two patients (one in each group) were predischarged from hospital on days 13 and 15 due to financial reasons. Except for the three patients who died within the first 4 days after the initiation of EN, all patients (100%) in the probiotic group and 80% of the patients in the control group were fed mainly via the enteral route by day 7 (*P *= 0.066). No patients stopped EN for more than 2 consecutive days. Thus, 43 patients successfully completed the 21-day study and were included in the per-protocol analysis (Figure 1). There were no differences in baseline characteristics between groups (Table 1). Intention-to-treat and per-protocol analyses yielded similar results and thus only results of the former are shown here.

**Table 1:** Patient characteristics and clinical outcomes

	Intention to treat		*P *value
		
	Probiotic (*n *= 26)	Control (*n *= 26)	
Age (years)	40.5 ± 13.0	40.8 ± 12.8	0.940
Gender (male/female)	19/7	21/5	0.510
Body mass index (kg/m^2^)	22.3 ± 1.1	22.3 ± 1.4	0.841
Glasgow Coma Scale	6.31 ± 1.01	6.42 ± 1.03	0.689
Blood glucose (mmol/l)	8.0 ± 1.1	7.9 ± 1.5	0.830
Patients using pantoprazole	20 (76.9%)	19 (73.1%)	0.749
Patients receiving MV < 48 hours	15 (57.7%)	16 (61.5)	0.777
Patients receiving MV ≥ 48 hours	1 (3.8%)	3 (11.5%)	0.574
APACHE II score			
Day 1	14.8 ± 3.6	14.3 ± 3.6	0.645
Day 4	14.2 ± 5.8	13.7 ± 5.5	0.753
Day 8	11.5 ± 6.4	12.8 ± 6.5	0.497
Day 15	9.2 ± 6.4	11.5 ± 7.9	0.305
Day 21	8.0 ± 7.7	9.9 ± 8.5	0.368
SOFA score			
Day 1	6.5 ± 1.4	6.3 ± 1.4	0.679
Day 4	5.7 ± 2.9	5.9 ± 2.2	0.341
Day 8	4.6 ± 3.3	5.1 ± 2.8	0.369
Day 15	3.7 ± 3.5	4.9 ± 4.4	0.374
Day 21	3.5 ± 4.2	4.4 ± 4.7	0.454
Duration of antibiotic use (days)	11.9 ± 4.9	14.1 ± 6.0	0.154
Types of antibiotics (*n*/day)	1.3 ± 0.5	1.7 ± 0.7	**0.021**
Length of ICU stay (days)	6.8 ± 3.8	10.7 ± 7.3	**0.034**
28-day mortality	3 (11.5%)	5 (19.2%)	0.701

Data presented as mean ± standard deviation or *n *(%). Significant differences in bold. MV, mechanical ventilation; APACHE, Acute Physiologic Chronic Health Evaluation; SOFA, Sequential Organ Failure Assessment.

### Immune variables

**Figure 2:**
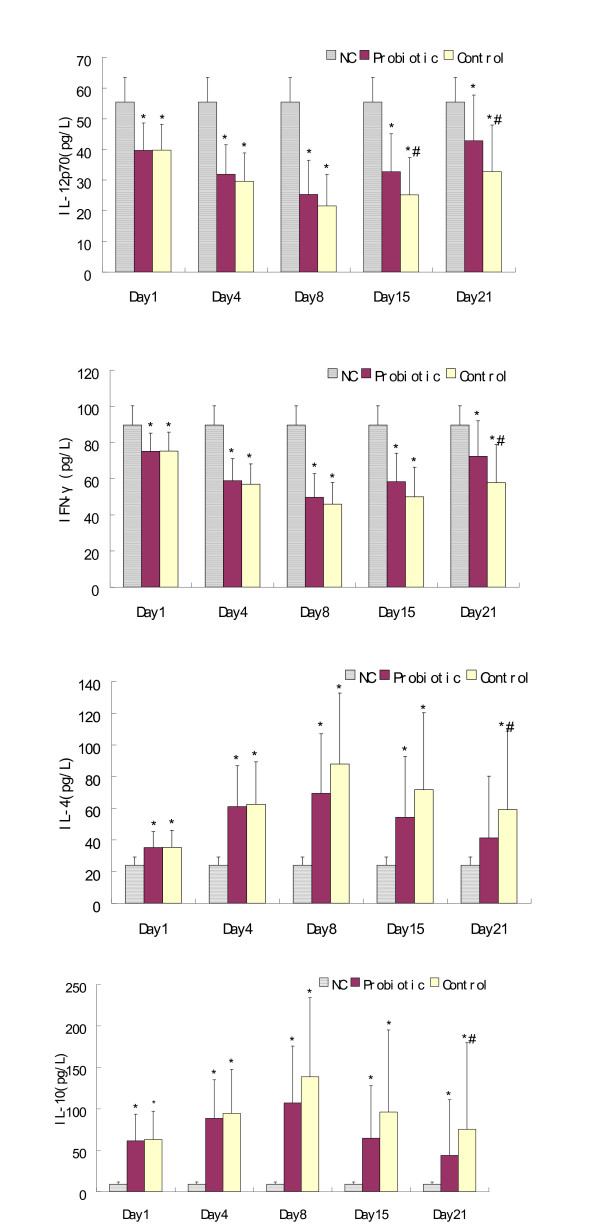
**Serum IL-12p70, IFNγ, IL-4, and IL-10 concentrations**. Mean ± standard deviation. *P < 0.05, compared with normal controls (NC). ^#^*P *< 0.05, compared with the probiotic group.

**Figure 3:**
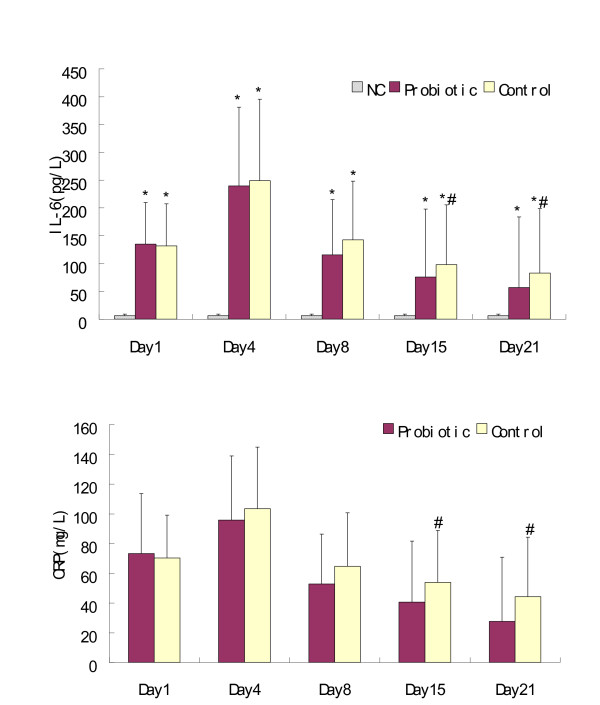
**Serum IL-6 and C-reactive protein concentrations**. Mean ± standard deviation. CRP, C-reactive protein. *P < 0.05, compared with normal controls (NC). ^#^*P *< 0.05, compared with the probiotic group.

As expected, the patients' serum levels of IL-12p70 and IFNγ were significantly lower than those of normal controls throughout the study period and reached a nadir on day 8, whereas the concentrations of IL-4 and IL-10 exhibited an opposite tendency. On day 15, however, the patients who received probiotics showed higher levels of IL-12p70 than did the control patients (*P *= 0.042). On day 21, significantly higher increases of both IL-12p70 and IFNγ were observed in the probiotic group compared with those of the control group (*P *= 0.023 and *P *= 0.017, respectively). Interestingly, the probiotic group exhibited significantly lower levels of IL-4 and IL-10 than the control group on day 21 (*P *= 0.017 and *P *= 0.027, respectively), and appeared to have similar IL-4 concentrations to the normal controls (*P *= 0.06). With respect to IL-6 and CRP, all patients demonstrated an early increase after injury and reached a peak on day 4 with the following decreasing tendencies: significantly lower levels were found among patients who received probiotics compared with patients in the control group on day 15 (*P *= 0.034 and *P *= 0.039, respectively) and on day 21 (*P *= 0.042 and *P *= 0.016, respectively) (Figures 2 and 3).

### Clinical outcomes

During the study period, 24 patients developed infections and no significant differences were found between groups (34.6% vs. 57.7%, *P *= 0.095). VAP was diagnosed in seven (43.8%) of the 16 patients in the probiotic group and in 13 (68.4%) of the 19 in the control group receiving endotracheal incubation and mechanical ventilation. In the other patients not mechanically ventilated, two in the probiotic group and one in the control group developed pneumonia. One of the 13 patients who acquired VAP and another one in the control group developed urinary tract infection (Table 2). The species of organisms isolated on tracheobronchial secretions cultures in patients with VAP are shown in Table 3. All patients acquired pathogens and presented VAP within 7 days after admission, yet eight of the 13 patients who developed VAP in the control group reacquired another one or two types of pathogens in the late period (> 7 days), while only one in the probiotic group experienced such conditions (61.5% vs. 14.3%, *P *= 0.07). As a result, more patients in the control group were infected by more than two types of pathogens compared with the probiotic group (76.9% vs. 14.3%, *P *= 0.017). Moreover, the two events of urinary tract infection occurred in the late period as well, presenting a higher late infection rate related to the absence of probiotics (38.5% vs. 4%, *P *= 0.002).

**Table 2:** Number and type of infections

	Probiotic group	Control group	*P *value
Type of infection			
Ventilator-associated pneumonia	7 (43.8%)	13 (68.4%)	0.182
Pneumonia^a^	2 (20%)	1 (16.7%)	1.000
Urinary tract infection	0	2 (7.7%)	0.471
Wound infection	0	0	
Bloodstream infection	0	0	
Patients with infections	9 (34.6%)	15 (57.7%)	0.095
Patients with one infection	9 (34.6%)	14 (53.8%)	
Patients with two infections	0	1 (3.8%)	

Data presented as *n *(%). ^a^Involved patients were not mechanically ventilated.

**Table 3:** Isolated pathogens on tracheobronchial secretions cultures in patients with ventilator-associated pneumonia

	Probiotic group (*n *= 16)^a^	Control group (*n *= 19)^b^	*P *value
Isolated pathogen			
*Pseudomonas aeruginosa*	5 (31.3%)	9 (47.4%)	0.491
*Staphylococcus aureus*	2 (12.5%)	5 (26.3%)	0.415
*Acinetobacter baumanii*	2 (12.5%)	6 (31.6%)	0.244
*Klebsiella pneumoniae*	0	2 (10.5%)	0.489
*Escherichia cloacae*	0	4 (21.1%)	0.109
Yeast-like fungus	0	2 (10.5%)	0.489
Patients with VAP infected with ≥ 2 kinds of pathogens	1 (14.3%)	10 (76.9%)	**0.017**
Patients reacquired another pathogen 7 days after admission	1 (14.3%)	8 (61.5%)	0.07

Data presented as *n *(%). Significant differences in bold. ^a^Sixteen patients in the probiotic group received mechanical ventilation. ^b^Nineteen patients in the control group received mechanical ventilation. VAP, ventilator-associated pneumonia.

All patients received antibiotic prophylaxis, and no significant difference was observed in terms of the days of antibiotic treatment between groups. However, patients in the control group were treated with significantly more types of antibiotics (Table 1). The antibiotics primarily used were cefmetazole, imipenem, and vancomycin.

There were no significant differences to the APACHE II or SOFA scores at any time point, but the length of ICU stay was significantly shorter among patients in the probiotic group (Table 1). No significant difference was found with regards to the 28-day mortality rate.

### Adverse events

No adverse effects of probiotic therapy were observed during the study period. No patients developed sepsis related to *Lactobacillus *and bowel ischemia.

## Discussion

The present study showed that the administration of probiotics could adjust the Th1/Th2 imbalance induced by severe TBI, and could result in a decreased infection rate during the late period, decrease the use of antibiotics, and result in a shorter stay in the ICU.

Previous studies indicated that probiotic therapy could reduce intestinal hyperpermeability, elevate IgA and IgG concentrations, decrease CRP, IL-6 and lipopolysaccharide levels in critically ill or multiple-injured patients [20-23], and skew the Th1/Th2 balance toward Th1 in allergic murine models and patients as well as antibiotic-treated mice by both *in vitro *and *in vivo *studies [24-27]. Our current study was the first randomized trial that investigated the effects of probiotics in severe TBI patients with the Th1/Th2 balance as a primary endpoint.

Literature has reported that the extent and duration of the Th1 suppression in brain-injured patients has a positive correlation with an increased risk of infectious complications and poor outcomes [2,4]. An early and long period of impaired cellular immune function, manifested by sustained low levels of IFNγ and high concentrations of IL-4 and IL-10, was shown in the present study. However, patients who received probiotics exhibited higher levels of IL-12 with a further Th2-to-Th1 switch compared with the controls after 15 days of treatment, and the differences were significantly demonstrated by day 21. The underlying mechanisms were unknown and not addressed in the current study, but may be related to the considerable DCs that are present in the gastrointestinal tract and play a critical role in the polarization of Th0 cells to Th1 or Th2 subsets by secreting IL-12, in which Toll-like receptors may be involved [28].

*In vitro *studies have suggested that probiotics could improve the maturation of DCs and further skew T cells to Th1 polarization [29,30]. However, such effects appear to vary among strains and species. For example, *Lactobacillus reuteri *upregulated surface mature makers on DCs but inhibited the production of IL-12 [30]. The probiotic cocktail VSL#3 - containing *Lactobacillus casei, Lactobacillus plantarum, Lactobacillus acidophilus, L. delbrueckii bulgaricus, Bifidobacterium breve, B. longum, Bifidobacterium infantis*, and *S. salivarius thermophilus *- induced the secretion of IL-10 by DCs from blood and intestinal tissue and restrained the generation of Th1 cells [31]. Probiotic effects thus cannot be generalized and the characteristics of individual probiotic strains need to be understood to pair them with specific disease states or desired goals. The probiotics we chose showed obvious Th1 polarization by *in vitro *and *in vivo *studies [26].

There were decreased tendencies regarding the total infections (34.6% vs. 57.7%, *P *= 0.095) and VAP (43.8% vs. 68.4%, *P *= 0.182) among patients in the probiotic group, but no significance was achieved. This could be primarily explained by the small sample size involved in our study. Based on the data we obtained, at least 60 mechanically ventilated patients would be required in each arm to obtain differences with regards to the incidence of VAP, with an 80% power at a risk of 0.05. One trial after the publication of the aforementioned two meta-analysis [10,11] involved 146 mechanically ventilated patients and showed significantly reduced VAP and fewer days of antibiotics prescribed for VAP with probiotic therapy [32]. However, another study failed to demonstrate a significant decrease in VAP with the presence of probiotics, but a difference was found relating to catheter-related bloodstream infections [33].

Our study showed no significant differences regarding the days of antibiotics use, but patients in the probiotic group were treated with fewer types of antibiotics. In addition, we found in the late period (7 days following admission) that fewer patients in the probiotic group acquired or reacquired pathogens. A significantly lower late infection rate was observed in the probiotic group (4% vs. 38.5%, *P *= 0.002), which coincided with the improved cellular immunity in the late period, suggesting a role for probiotics in inhibiting the invasion of pathogenic bacteria and suppressing infections in the severely brain-injured patient. Similar findings were observed in previous reports by Knight and Fukushima. In the study conducted by Knight and colleagues, a decreased incidence of VAP was found (9% vs. 13%) in the symbiotic group - which, however, exhibited a slightly higher colonization rate by pathogens compared with the control group [34]. In the other study, patients who received probiotics demonstrated a significantly lower infection rate and more improved immunity than those of the control group but without any significant changes in fecal microbiota between groups at any time point [35]. One may thus postulate that probiotics may act themselves mainly by enhancing the immunological status.

CRP is an extremely sensitive marker of inflammation that can differentiate the various stages of systemic inflammatory response syndrome in the absence of an infection. Combined with another important inflammatory cytokine (IL-6), CRP correlates positively with illness severity and can be used to predict subsequent organ dysfunction and mortality [36]. As was seen in the previous studies [20-22], the present study also demonstrated lower serum levels of CRP and IL-6 in the probiotic group than those of the control group by days 15 and 21 (Figure 3). This may be partially attributable to more patients in the control group acquiring infections during the late period.

A shorter length of ICU stay was observed with probiotic therapy, but the APACHE II and SOFA scores as well as the 28-day mortality rate revealed no significant differences (Table 1). The small sample size may be one main reason contributing to these results. Similar findings were also illustrated in other studies [20,21], in which 28 and 72 ICU patients were recruited in total, and probiotics increased the serum levels of IgA and IgG, and decreased the rate of VAP without significant improvement of multiple organ dysfunction syndrome scores and mortality rates.

Our study indicated that no adverse events were linked to probiotic treatment as reported by Besselink and colleagues [37]. A recent systematic review concluded that the majority of probiotics can be safely used in patients receiving nutritional support, as per the evidence found in another 72 articles, including that of Besselink and colleagues. Only three trials showed increased complications among specific patients who experienced transplant and pancreatitis, and two of the three patients received probiotics through a postpyloric tube [38].

The probiotics we chose for this study - *B. longum, L. bulgaricus*, and *S. thermophilus *- are well known for their safety. The *Bifidobacteria *belong to the largest anaerobic bacterial populations of the human gastrointestinal tract. *In vitro *studies showed that *B. longum *was not only able to grow in 2% bovine bile, grow at pH 3.5, inhibit most intestinal pathogens, produce bacteriocin-like substances, and adhere to human epithelial cells, but also showed no resistance to antibiotics [39]. In addition, by means of reducing the redox potential to sufficient levels and manufacturing lactic acid, *L. bulgaricus *can provide a good environment for the growth of *B. longum *[40].

Despite our findings, this research has several limitations and should be considered a pilot study. The small sample size and the single center studied, which carries inherent biases related to some local practice habits, limit the extrapolation of the results. Larger and multicenter studies are further required to test the effect of probiotics on these clinical outcomes. Our primary aim was to evaluate the changes of Th1/Th2 cytokines, however, and the data we achieved confirmed the effectiveness of probiotics and provided a basis for calculating the number of patients needed in future studies. The second limitation could be that the study was not double-blinded and placebo-controlled, because probiotic therapy must be prescribed by the physicians in charge after randomization. Furthermore, probiotics are readily available and may be prescribed by physicians to manage diarrhea in the research unit, so the investigator must rigorously monitor the study period to avoid such interference. As far as the absence of placebo is concerned, the involved patients were all unconscious and unaware of the trial. The routinely used semiquantitative cultures of tracheal aspirates used in the hospital to confirm a clinical diagnose of VAP may have been another limitation, as this has reduced accuracy in comparison with quantitative results. However, quantitative methods may not have been reliable in severely ill patients receiving prior antibiotics, for they exhibited markedly low sensitivity under these circumstances [41].

## Conclusions

Our findings suggest that daily prophylactic administration of probiotics could attenuate the deviated Th1/Th2 response induced by severe TBI, which may contribute to the decreased nosocomial infection rate, especially as shown in the late period.

## Key messages

• When administered enterally to severe traumatic injury patients, probiotics improved cellular immune function as illustrated by the Th2-to-Th1 switch in the late period.

• Probiotic therapy appears to be an alternative way to reduce the occurrence of nosocomial infections.

• Probiotics contribute to decreasing infections mainly by enhancing the immunological status.

• Probiotics could be safely used in critically ill patients.

## Abbreviations

APACHE: Acute Physiology and Chronic Health Evaluation; CRP: C-reactive protein; DC: dendritic cell; ELISA: enzyme-linked immunosorbent assay; EN: enteral nutrition; IFN: interferon; IL: interleukin; SOFA: Sequential Organ Failure Assessment; TBI: traumatic brain injury; Th: T-helper type cell; VAP: ventilator-associated pneumonia.

## Competing interests

The authors declare that they have no competing interests.

## Authors' contributions

MT designed and carried out the study, performed the statistical analysis, and drafted the manuscript. J-CZ conceived of the study, participated in its design, and helped to draft the manuscript. JD assisted with the statistical analysis and discussion. L-MZ participated in collecting and entering the data, and performed some of the statistical analysis. H-HY helped to plan the study. All authors read and approved the final manuscript.
